# Advancements in the treatment of non-alcoholic fatty liver disease (NAFLD)

**DOI:** 10.3389/fendo.2022.1087260

**Published:** 2023-01-16

**Authors:** Li Rong, Junyan Zou, Wei Ran, Xiaohong Qi, Yaokai Chen, Hongjuan Cui, Jinjun Guo

**Affiliations:** ^1^ Department of Gastroenterology, Bishan Hospital of Chongqing Medical University, Bishan Hospital of Chongqing, Chongqing, China; ^2^ Medical Research Institute, Southwest University, Chongqing, China; ^3^ Medical Research Institute, Southwest University, Public Health Hospital Affiliated to Southwest University, Chongqing, China; ^4^ Department of General surgery, Baoshan People’s Hospital of Yunnan Province, Baoshan, Yunnan, China

**Keywords:** nonalcoholic fatty liver disease, non-alcoholic steatohepatitis, diet, exercise, medical treatment

## Abstract

Non-alcoholic fatty liver disease (NAFLD) is a series of diseases, involving excessive lipid deposition in the liver and is often accompanied by obesity, diabetes, dyslipidemia, abnormal blood pressure, and other metabolic disorders. In order to more accurately reflect its pathogenesis, an international consensus renamed NAFLD in 2020 as metabolic (dysfunction) associated with fatty liver disease (MAFLD). The changes in diet and lifestyle are recognized the non-drug treatment strategies; however, due to the complex pathogenesis of NAFLD, the current drug therapies are mainly focused on its pathogenic factors, key links of pathogenesis, and related metabolic disorders as targets. There is still a lack of specific drugs. In clinical studies, the common NAFLD treatments include the regulation of glucose and lipid metabolism to protect the liver and anti-inflammation. The NAFLD treatments based on the enterohepatic axis, targeting gut microbiota, are gradually emerging, and various new metabolism-regulating drugs are also under clinical development. Therefore, this review article has comprehensively discussed the research advancements in NAFLD treatment in recent years.

## 1 Introduction

With the deepening of the research on the mechanism of Non-alcoholic fatty liver disease(NAFLD), the two-hit theory cannot fully explain interactions between genetics and environment as well as those between different organ systems; therefore, the multi-hit theory has gradually been accepted (1). Currently, NAFLD is considered a complex metabolic disease, resulting from the complex interactions among genetic susceptibility, host metabolic disorders, and environmental factors. Numerous factors affect its clinical manifestations and disease progression, including race, genetic susceptibility, dietary habits, metabolism, immunity, gut microbiota, and other factors. Insulin resistance is one of the recognized mechanisms of NAFLD. However, the interaction between lipid metabolism disorder and its triggered inflammatory response can jointly promote the occurrence and development of NAFLD. In recent years, numerous genome-wide studies on NAFLD identified multiple polymorphic changes in the genes associated with the susceptibility to NAFLD, suggesting that both the genetic and epigenetic changes might play an important role in the pathogenesis of NAFLD. The abnormal immune microenvironment of the liver and its mediated inflammatory response might also play an important role in the pathogenesis of NAFLD. More studies showed that the changes in the composition and structure of gut microbiota might also have an important impact on the occurrence and development of NAFLD. The incidence of NAFLD is continuously increasing rapidly worldwide and has become the primary cause of chronic liver diseases in the world. Therefore, the studies and development of targeted drugs based on the above-mentioned important links in the pathogenesis of NAFLD are also in full swing. Therefore, it is necessary to have a systematic understanding of the current treatment methods and drugs for NAFLD in order to provide a certain reference and help effectively improve the clinical prognosis of NAFLD patients.

## 2 Treatment strategies of NAFLD

Although the exact pathogenesis of NAFLD remains to be elucidated, the associated risk factors are clear and include unhealthy lifestyle, insulin resistance (IR), type 2 diabetes mellitus (T2DM), increased liver lipogenesis, and dysregulation of gut microbiota. There is currently no standard treatment for NAFLD; however, the clinical practice guidelines of the European Association for the Study of Liver (EASL) -European Association for the Study of Diabetes (EASD), European Association for the Study of Obesity (EASO) (2) and the American Association for the Study of Liver Diseases(AASLD) Practice Guidelines (3) clearly state that the lifestyle changes can lead to 5-10% weight loss in the overweight/obese NAFLD patients. Furthermore, the National Institute for Health and Care Excellence (NICE) guidelines (4) also recommend changes in lifestyle as the first choice of treatment for NAFLD patients. Therefore, optimizing the lifestyle through a rational diet and exercise intervention is undoubtedly the basis and important link of NAFLD treatment. The combination of drugs, which can effectively regulate glucose and lipid metabolism and reduce liver inflammation and fibrosis, might be more beneficial for the treatment of NAFLD.

### 2.1 Non-drug treatment strategies

#### 2.1.1 Dietary changes

Severely restricting total calories, inducing ketosis, or reducing free sugar intake and limiting carbohydrate consumption can enhance liver protection, thereby making the dietary interventions a promising strategy for the treatment of NAFLD (5). Caloric restriction (CR) is the most common dietary intervention treatment strategy for NAFLD.

A meta-analysis found that western dietary patterns could increase the risk of NAFLD by 56%, indicating that dietary pattern was associated with the risk of NAFLD, whereas a Mediterranean diet (MD) could reduce this risk by 23% (6). MD is defined as a plant-based diet characterized by a high proportion of monounsaturated fatty acids (MUFA) and saturated fatty acids (SFA), in which, the total fat accounts for 30-40% of the daily energy consumption (7), and is based on fruits and vegetables, fish, whole grains, legumes, and olive oil (8). The EASL-EASD-EASO Clinical Practice Guidelines recommend MD as an optional diet for the treatment of NAFLD because MD can improve metabolism by reducing IR and lipid concentrations and induce the regression of steatosis as well as significantly reduce the cardiovascular events (1, 2, 7, 9, 10). Therefore, this might be an effective and safe approach for the treatment of patients with MS and NAFLD (11). MD could improve overweight and visceral obesity, reduce hepatic steatosis, and reduce liver cirrhosis in NAFLD patients (12–14). It could also improve IR, which in turn improved the disease condition of NAFLD patients (7, 15). A meta-analysis by Japanese scholar Takumi Kawaguchi also confirmed this view that MD could improve hepatic steatosis and IR in patients with NAFLD (16). Recently, clinical trials have provided evidence of MD feasibility in adolescents and children with NAFLD. In children aged 9-17 years with NAFLD, the 12-week administration of MD could improve IR (17). Similarly, this diet plan could reduce the body mass index (BMI), fat mass, and hepatic steatosis, improve IR, and reduce the levels of transaminases, inflammatory markers, and oxidative stress markers in children aged 11-18 years with NAFLD (18).

In addition to MD, the ketogenic diet (KD) has also been used as a dietary intervention for the treatment of NAFLD in recent years. KD has a high proportion of fats and a low proportion of carbohydrates, proteins, and other nutrients, and due to its extremely low proportion of carbohydrates, it also plays a positive role in the treatment of NAFLD (19). It can significantly alter the mitochondrial flux and redox status of the liver to promote ketogenesis without affecting the synthesis of intrahepatic triglycerides (IHTG), thereby significantly improving the visceral fat contents and IR (20). Although the above studies suggest that KD has certain therapeutic effects on NAFLD patients, several animal and clinical trials have indicated its risk. An animal study showed that the high-fat diet (HFD)-induced obese or NAFLD male C57Bl/6NJ mice were likely to develop hepatic mitochondrial dysfunction after the long-term intervention of KD (21). A case study has also reported the risks of acutely-worsened hyperlipidemia and elevated liver enzymes associated with a KD (22). Therefore, based on the current clinical research results, the safety of KD for the treatment of NALFD requires further investigations.

In addition, several new dietary interventions have been used for the gradual treatment of NAFLD patients. For example, a low-calorie and low-fat diet could significantly reduce the liver fat contents (LFCs) in NAFLD patients in the short term (23). A high-protein (animal or plant) diet could also significantly reduce the LFC, IR, and levels of inflammatory markers (24). An eight-week free sugar restriction diet could reduce liver fat (25) and improve liver steatosis in adolescent boys with NAFLD (26). However, due to the limited number of clinical trials based on these dietary interventions, they have not yet drawn sufficient conclusions. Similarly, there is limited clinical data and unclear results for the interventions of very low-calorie KD (VLCKD), intermittent feeding (IF), and time-restricted feeding (TRF); Therefore, their safety and effectiveness cannot be verified currently.

#### 2.1.2 Exercise intervention

In addition to dietary intervention, exercise can also improve glucose and lipid metabolism disorders (27) and reduce LFC (28) and is an effective method for the treatment of metabolic-related diseases. Regular exercise can effectively treat NAFLD in both non-obese and obese patients (29), and the combination of resistance exercise and aerobic exercise has been proved to be more reasonable and effective in clinical practice. For instance, resistance exercise is relatively safe and could effectively improve the metabolic status of NAFLD patients (30). A 12-week resistance exercise, consisting of push-ups and squats, could help in the prevention of NAFLD progression (31). Aerobic exercise also showed similar effects. A 12-week aerobic exercise intervention could improve liver fibrosis (32). Furthermore, the high-intensity interval (HII) exercise for 12 weeks could reduce the blood glucose contents and waist circumference in NAFLD patients (33). The individually designed eight-week exercise program could reduce liver steatosis, the level of inflammatory markers including hypersensitivity C-reactive protein (hs-CRP), and ferritin, and reduce fibrosis (34). However, studies have shown that there was no significant difference in the effects of the different amounts or intensities of aerobic exercise on the LFC; the 8 weeks of low-medium-intensity high-volume aerobic exercise, moderate-to-low-intensity low-volume aerobic exercise, and high-intensity low-volume aerobic exercise could reduce LFC and visceral adipose tissue (VAT) with insignificant differences between them (35). Both the HII and moderate-intensity continuous (MIC) aerobic exercise for 8 weeks could reduce IHTG and visceral fats in obese NAFLD patients with T2DM (36).

The sample size of these clinical studies on exercise intervention was relatively small, and most of the researchers only focused on observing the effects of an exercise intervention on certain body indices, such as BMI, blood sugar, LFC, etc., in patients with NAFLD (37). At the same time, most relevant studies did not focus on the benefits of an exercise intervention on liver fibrosis in NAFLD patients. Therefore, there is currently insufficient evidence that these interventions might bring definite benefits to patients with NAFLD.

#### 2.1.3 Diet and exercise interventions

The methods of combining exercise with caloric restriction might affect NAFLD by increasing energy expenditure, reducing lipid overload, and improving metabolic homeostasis (15). This dual intervention approach might have better effects both in animal and clinical studies. Exercise and changing the diet from an HFD to a regular diet could alleviate HFD-induced hepatic steatosis in Sprague Dawley (SD) rats (38).

In a randomized controlled trial, the combination of exercise with a green-MD (a diet with a limited amount of red/processed meat and an increased proportion of green plants and polyphenols in MD) could further reduce intrahepatic fat contents and NAFLD prevalence as compared to normal diet and MD (39). Similarly, a randomized controlled trial (40) reported that exercise and dietary interventions could improve LFC and glucose metabolism in NAFLD patients. These studies suggested that the combination of exercise with dietary intervention might have a better effect on NAFLD patients (41). Therefore, for obese patients with NAFLD, who have time and energy, a combination of exercise and diet interventions should be prioritized.

#### 2.1.4 Bariatric surgery

Bariatric surgery is an option for obese patients with NAFLD, who are non-responsive to dietary changes and exercise or unable to lose weight through lifestyle changes. Bariatric surgery is another common method for the treatment of obesity in the United States and European countries, but it is relatively rare in China. The American Society for Metabolic and Bariatric Surgery Pediatric Committee (ASMBS) recommends metabolic and bariatric surgery (MBS) as an effective treatment for severe obesity in adolescents and should be considered the standard of care (42). Similarly, the AALSD practice guidelines also recommend that foregut bariatric surgery might be considered in obese patients with NAFLD or NASH (43). Bariatric surgery can improve liver histology, including fibrosis, which is secondary to NASH. In addition, bariatric surgery has other benefits, including improvement or remission of T2DM, dyslipidemia, and hypertension, and reduction of cardiovascular disease (CVD)’s morbidity or mortality (44). Bariatric surgery could also significantly improve the BMI and liver fibrosis scores (45) as well as all the histological features of NAFLD, including fibrosis (2, 3, 46).

As any surgical treatment has risks, bariatric surgery is also not completely safe and effective. The condition of a NAFLD patient deteriorated after bariatric surgery. Although the patient lost 35% of his body weight rapidly, he developed acute liver failure as a serious complication (47). There are high risks of postoperative complications in patients with cirrhosis reportedly, while in well-compensated cirrhotic patients, it might be used as an aid to improve the long-term outcome (48). Overall, the clinical efficacy and safety of bariatric surgery require further investigations to make it suitable for NAFLD patients.

### 2.2 Medical treatment

#### 2.2.1 T2DM drugs

Although the exact pathogenesis of NAFLD is still unclear, IR plays a key role in the development of NAFLD. The prevalence of NAFLD is high in T2DM patients; it is closely associated with obesity, hyper-glycated hemoglobin, hyperlipidemia, and hyper-alanine aminotransferase (hyper-ALT) (49). Therefore, antidiabetic drugs are often used for the clinical treatment of T2DM patients with NAFLD.

##### 2.2.1.1 Glucagon-like peptide-1 receptor agonists

GLP-1, an intestinal hormone released from the foregut in response to food consumption, can stimulate insulin secretion and inhibit glucagon secretion. GLP-1RAs, such as liraglutide, semaglutide, dulaglutide, exenatide, etc., are new antidiabetics. Studies showed that these drugs could reduce body weight and improve IR as well as improve liver enzyme levels and reduce LFC in T2DM patients; this might be beneficial for the treatment of NAFLD/NASH patients (50). Therefore, these drugs have often been used in clinical studies of NAFLD in recent years.

Liraglutide has been the most widely studied GLP-1RA drug in NAFLD (51), which can improve IR (52) and reduce LFC in T2DM patients (53). A Phase II clinical trial showed that the subcutaneous injection of liraglutide at a dose of 1.8 mg/d in NASH patients could improve the histopathology of the liver, which was manifested as regression of NASH, patient’s well-tolerance, and no progression of liver fibrosis (54). In a study by Feng et al., the treatment effects of liraglutide on T2DM patients with NAFLD were better than that of metformin and gliclazide (55), mainly in reducing the body weight, waist circumference, and intrahepatic fat contents. However, in terms of safety, as shown in clinical trials, the use of liraglutide is often associated with some mild gastrointestinal adverse reactions, including diarrhea, constipation, loss of appetite, etc. (54).

A study by Philip Newsome’s team found that obesity and T2DM were the drivers of NAFLD, and semaglutide could significantly reduce plasma ALT and hs-CRP levels in obese patients with T2DM; this demonstrated the potential of semaglutide for alleviating NAFLD (56). Then, they launched a randomized, placebo-controlled phase II clinical trial to investigate the clinical efficacy of semaglutide by subcutaneously injecting its different doses into patients with NASH. After 72 weeks of treatment, NSAH in the experimental group was relieved, which was mainly reflected in the reduction of liver inflammatory cells and liver cell degeneration; moreover, the proportion of patients with no further liver fibrosis was significantly higher than that in the control group. The optimal injection concentration obtained from the clinical trial was 0.4 mg/d, which showed the best effects. However, semaglutide could not significantly improve the degree of liver fibrosis in the patients (57). Currently, they are planning a phase III clinical trial to further validate the clinical efficacy of semaglutide in improving metabolic disorders (58). A study by Anne Flint’s team also showed that as compared to placebo, the subcutaneous injection of semaglutide at a dose of 0.4 mg/d could reduce LFC and improve liver enzyme levels; however, the effects on remission of liver cirrhosis were not obvious (59). Similar to liraglutide, semaglutide has some gastrointestinal side effects, among which, nausea is the most common (57, 60).

The subcutaneous injection of dulaglutide at a dose of 1.5 mg/week could reduce plasma ALT, aspartate aminotransferase (AST), and γ-glutamyl transpeptidase (GGT) levels in the patients with T2DM (61). Therefore, this drug could be applied for alleviating NAFLD. As compared to liraglutide, dulaglutide had the advantage of less frequent injections, usually once a week. A study by Kuchay et al. showed that the subcutaneous injection of dulaglutide at a dose of 1.5 mg/week could significantly reduce LFC and GGT in patients with T2DM and NAFLD without serious adverse reactions. Consequently, they suggested that dulaglutide could be used for the early treatment of T2DM patients with NAFLD (62). Similarly, a study conducted in Japan also showed that the subcutaneous injection of dulaglutide at a dose of 0.75 mg/week for 12 weeks could reduce body weight and improve liver enzyme levels in T2DM patients with NAFLD (63).

An early study reported that exenatide could improve blood glucose, body weight, and liver enzyme levels in T2DM patients with NAFLD (64). Due to the good liver protecting effects of exenatide, it was more suitable for NAFLD patients with obesity, elevated liver enzymes, and T2DM (65). A recent study by Liu et al. showed that the subcutaneous injection of exenatide at a dose of 10 μg twice a day could also reduce LFC in T2DM patients with NAFLD (66). A study conducted in Turkey also confirmed that the same dose of exenatide treatment could reduce blood sugar, reduce body weight, and improve liver fibrosis scores in T2DM patients with NAFLD (67). Gastaldelli et al. showed that the combination of exenatide and dapagliflozin was more effective as compared to their single drug treatment in the T2DM patients with NAFLD; this provided a novel treatment idea for the use of clinical drugs; however, more prospective trials are needed to verify these results (68).

Overall, most of the clinical studies conducted on using GLP-1RA drugs for the treatment of T2DM complicated with NAFLD or NASH showed that these drugs were safe and effective (**Table 1**), and the most common side effects included some gastrointestinal adverse reactions, such as diarrhea, nausea, vomiting, constipation, loss of appetite, etc. However, numerous prospective studies are needed to explore the reasonable dose and duration of these drugs.

**Table 1:** Effect of GLP-1RAs in patients of NAFLD/NASH: Data from clinical studies.

GLP-1RAs	Types	Treatment duration	Population	Effects	Adverse events	References
Liraglutide (1.8 mg/day)	multicentre, double-blind, randomised, placebo-controlled phase 2 study	48 weeks	NASH (overweight)	well tolerated and histological resolution of NASH	diarrhoea, constipation, loss of appetite	Armstrong et al. (54)
Semaglutide (0.1/0.2/0.4 mg/day)	placebo-Controlled, double-blind phase 2 trial	72 weeks	NASH	NASH resolution, 0.4mg/day (the best)	nausea, constipation, and vomiting	Newsome et al. (57)
Semaglutide (0.4 mg/day)	randomised, double-blind, placebo-controlled trial	72 weeks	NAFLD	resolution of liver steatosis and improvement in liver enzymes and metabolic parameters	diarrhoea, nausea, appetite decrease	Flint et al. (59)
Dulaglutide (1.5 mg/week)	open-label, parallel-group, randomised controlled trial	24 weeks	T2DM with NAFLD	LFC decrease and GGT levels improvement	upper gastrointestinal upset	Kuchay et al. (62)
Dulaglutide (0.75 mg/week)	retrospective study	12 weeks	T2DM with NAFLD	weight loss, liver enzymes improvement	diarrhea	Seko et al. (63)
Exenatide (10µg twice daily)	multicenter randomized controlled trial (insulin glargine)	24 weeks	T2DM with NAFLD	LFC decrease, weight loss, reduces in visceral fat area, FIB-4, postprandial plasma glucose, and LDL-C	hypoglycemic	Liu et al. (66)

##### 2.2.1.2 Sodium-glucose cotransporter 2 inhibitors

SGLT2i is a new type of oral antidiabetic drug, which can inhibit the reabsorption of glucose by the kidneys, thereby reducing blood glucose levels. A retrospective analysis found that SGLT2i could improve steatosis and fibrosis in patients with T2DM, therefore, they might be beneficial for the NAFLD patients (69). Japanese researchers conducted a five-year follow-up study on six T2DM patients with NAFLD and showed that three patients had improved liver histology after SGLT2i treatment (70). An 18-month randomized study by Korean researchers suggested that adding SGLT2i to common anti-sugar therapy in T2DM patients could better reduce fat contents and improve liver enzyme levels (71). A meta-analysis, summarizing 10 clinical trials, indicated that drug therapy with SGLT2i could improve LFC, liver enzyme levels, BMI, inflammatory markers, etc. in Asian T2DM patients with NAFLD (72). Likewise, another systematic review also confirmed the ameliorating effects of SGLT2i on LFC and liver enzyme levels (73).

Clinical trials have also proved the above point of view. Gaborit et al. suggested that the oral administration of empaglifozin (EMPA) at a dose of 30 mg/kg/day for 12 weeks in the C57BL/6 mice could reduce LFC, while the oral administration of EMPA at a dose of 10 mg/day for 12 weeks in T2DM patients could also reduce LFC effectively (74). Coincidentally, a randomized double-blind placebo-controlled phase IV clinical trial showed that the oral administration of EMPA at a dose of 25 mg/day for 24 weeks could effectively control blood sugar and reduce LFC in patients with T2DM (75). Recently, another trial in T2DM patients with CVD showed that either 10 or 25 mg/d EMPA treatment could reduce steatosis, but not liver fibrosis risk (76). Furthermore, in NAFLD patients without T2DM, the oral administration of 10 mg/d EMPA for 24 weeks could also improve hepatic steatosis and fibrosis (77).

Similarly, the oral administration of ipragliflozin at a dose of 50 mg/day for 24 weeks could improve liver enzyme levels and fasting blood glucose levels in T2DM patients with NAFLD (78). Japanese researchers reported similar findings (79). In another multicenter, open-label randomized controlled trial, a treatment regimen of 72 weeks with ipragliflozin could improve liver fibrosis, blood sugar levels, and obesity in NAFLD patients, and none of the patients developed NASH compared as compared to controls; therefore, ipragliflozin might play a role in the prevention and treatment of NASH (80).

In a double-blind, controlled, randomized trial, the oral administration of dapagliflozin at a dose of 10 mg/day for 8 weeks reduced serum aminotransferase levels and LFC in the patients with T2DM, which was beneficial to the liver (81). When applied to the T2DM patients with NAFLD, and the treatment regimen was extended to 12 weeks, dapagliflozin reduced the levels of hepatocyte injury biomarkers, including ALT, AST, GGT, etc., in patients, indicating an improving effect of dapagliflozin on NAFLD (82). A prospective study by Japanese researchers showed that in addition to the local standard of care for T2DM, the oral administration of dapagliflozin at a dose of 5 mg/day for 24 weeks could improve hepatic steatosis in T2DM patients with NAFLD and attenuate hepatic fibrosis in patients with significant hepatic fibrosis (83). As for the efficacy of dapagliflozin in NAFLD patients without T2DM, another prospective study by Japanese researchers, including 12 patients, showed that the oral administration of dapagliflozin at a dose of 5 mg/d for 12 weeks could reduce weight and improve liver enzymes (84).

There are relatively few studies on the administration of canagliflozin. The oral administration of canagliflozin at a dose of 100 mg/day for 24 weeks could improve liver function and reduce visceral fat, thereby exhibiting therapeutic potential for T2DM patients with NAFLD (85). However, this study included only five patients. Another retrospective study had the same limitation. A study of 7 patients suggested that the long-term treatment with canagliflozin at a dose of 100 mg/d could improve liver histology in six patients and worsen it in one patient (86). Therefore, further studies are needed to discover the efficacy of canagliflozin.

To sum up, in clinical studies, most of the SGLT2i drugs are applied to T2DM patients with NAFLD (**Table 2**). Although there are fewer clinical studies on the applications of these drugs to NAFLD patients without T2DM, they have good application prospects. The safety of drugs should also be investigated. SGLT2i has several adverse reactions, such as hypoglycemia, ketoacidosis, and urinary tract and genital infections, when used for the treatment of T2DM patients (87). As a result, their clinical use in patients with NAFLD requires more exploration.

**Table 2:** Effect of SGLT2i in patients of NAFLD/NASH: Data from clinical studies.

SGLT2i	Types	Treatment duration	Population	Effects	Adverse events	References
Empaglifozin (10mg/day)	randomized, double-Blind, placebo-controlled Trial	24 weeks	NAFLD	improvement in liver steatosis and fibrosis	mild fungal vaginal infections and allergic reactions	Taheri et al. (70)
Empaglifozin (10mg/day)	randomized, double-Blind, phase 4, placebo-controlled Trial	24 weeks	T2DM with NAFLD	improvement in liver steatosis and fibrosis	no mention	Kahl et al. (76)
Ipragliflozin (50mg/day)	randomized, open-label, active-controlled trial (Pioglitazone 15-30 mg/day)	24 weeks	NAFLD	weight loss and abdominal fat area decrease	urinary tract infections, vaginal candidiasis	Ito et al. (78)
Ipragliflozin (50mg/day)	prospective study	24 weeks	T2DM with NAFLD/NASH	weight loss, improvement in liver enzymes, body fat mass and steatosis	no mention	Miyake et al. (79)
Ipragliflozin (50mg/day)	multicenter, open-label, randomized controlled trial	72 weeks	T2DM with NAFLD	improvement in liver fibrosis, blood sugar levels and obesity	frequent urination, increase in HbA1c	Takahashi et.al. (80)
Dapagliflozin (10mg/day) alone or combination of omega-3 (n-3) carboxylic acids (4g/day)	multicenter, double-blind, randomized, placebo-controlled trail	12 weeks	T2DM with NAFLD	biomarkers of liver cell injury decrease	mild or moderate in intensity	Eriksson et al. (82)
Dapagliflozin (5mg/day)	prospective, randomized, active-controlled, open-label trial	24 weeks	T2DM with NAFLD	improvement in liver steatosis and fibrosis	no mention	Shimizu et al. (83)
Dapagliflozin (5mg/day)	single center, double-blind, randomized, prospective study	12 weeks	NAFLD	weight loss, improvement in liver enzymes	NA	Tobita et al. (84)
Canagliflozin (100mg/day)	prospective, single center, single arm, nonrandomized, open, and uncontrolled clinical trial	24 weeks	T2DM with NAFLD	improvement in liver function and visceral fat	no mention	Akuta et al. (85)

##### 2.2.1.3 Biguanides

Biguanides are very effective in the treatment of T2DM. Metformin, a common antidiabetic drug, is an example of biguanides. It can reduce endogenous glucose production, activate AMPK, and inhibit mitochondrial glycerophosphate dehydrogenase (88); however, its safety and efficacy in NAFLD patients are still controversial (89). In an animal experiment, the administration of 300 mg/kg/d metformin in diet could reduce the occurrence of NAFLD in C57Bl/6J mice as compared to the high-sugar and high-fat diets alone (90). However, unlike animal experiments, metformin did not show the same effects in clinical studies. Metformin showed modest improvement in body weight, waist circumference, and liver enzyme levels after 24 weeks of long-term treatment in T2DM patients with NAFLD (55). Similarly, a systematic review and meta-analysis demonstrated that metformin treatment could not improve liver histopathology in NASH patients (91). In recent years, its effectiveness has been controversial. Japanese researchers showed that increasing the dose of metformin to 1500 mg 2-3 times/d for 52 weeks could reduce hepatic steatosis in T2DM patients with NAFLD (92). A study by Mitrovic et al. demonstrated that metformin could also reduce the levels of hs-CRP, ferritin, and other inflammatory factors in non-obese T2DM patients with NAFLD (93). In addition, some clinical trials investigated the concomitant effects of metformin with GLP-1RA or SGLT2i in T2DM patients with NAFLD; however, their effects were not satisfactory. For example, a 24-week prospective randomized placebo-controlled trial by Harreiter et al. showed that metformin combined with exenatide or dapagliflozin did not show additive effects on hepatocyte lipid reduction (94). Overall, there is insufficient evidence that Metformin has a definite effect in the treatment of NAFLD; however, some studies suggest that Metformin might play a role in the prevention of hepatocellular carcinoma and tumors (95), which requires further studies.

##### 2.2.1.4 Thiazolidinediones

Thiazolidinediones act as peroxisome proliferator-activating receptor PPAR-γ agonists that improve IR in obese and concomitant type 2 diabetes mellitus. Pioglitazone is the only diabetic agent incorporated into the treatment guidelines for NASH (2, 3, 96). A randomized controlled trial showed that the oral administration of metformin at a dose of 2g/d combined with a low dose (mean 26 mg/d) of pioglitazone for one year could improve liver steatosis, inflammation, and IR in the T2DM patients with NAFLD (97). The effectiveness of pioglitazone alone was investigated in a placebo-controlled study. The oral administration of pioglitazone at a dose of 30 mg/d for 96 weeks could improve liver histology, liver enzyme levels, and IR in non-diabetic NASH patients (98). Similarly, in a study by Aithal et al., a treatment regimen with the same dose for 12 months could improve liver metabolic and histological parameters, most notably in the level of liver injury and fibrosis in NASH patients (99). The restricted dietary interventions combined with pioglitazone therapy might also improve liver metabolism in NASH patients. In a study by Renata Belfort et al., a low-calorie diet (500-kcal/d deficit from weight-maintaining caloric intake) combined with 45 mg/d pioglitazone for 6 months could improve metabolism and liver histology in the NASH patients (100). Similar results were also demonstrated by Kenneth Cusi et al. A treatment regimen with the same intervention conditions described above for 18 months was safe and effective in the patients with prediabetes or T2DM and NASH as well as improved their liver histology without worsening fibrosis (71). However, pioglitazone is not suitable for all patients. It might aggravate lower extremity edema; therefore, caution should be taken in patients with severe obesity, diastolic dysfunction, congestive heart failure, concomitant use of amlodipine or high-dose insulin, etc (96).

In general, the antidiabetic drugs used to alleviate NAFLD have certain clinical effects and research potential. Relevant guidelines have also recommended some antidiabetic drugs for the clinical treatment of NAFLD.

#### 2.2.2 Lipid-lowering drugs

Statins, common clinical lipid-lowering drugs, can inhibit cholesterol synthesis by blocking 3-hydroxy-3-methyl glutaryl coenzyme A (HMG-CoA) reductase (101). Dyslipidemia in NAFLD patients is typically characterized by elevated triglyceride (TG) and low-density lipoprotein cholesterol (LDL-C) levels and reduced high-density lipoprotein cholesterol (HDL-C) levels, and the deposition of hepatic cholesterol also contributes to the progression of NAFLD (102, 103). A meta-analysis by Kim et al. showed that statins could reduce the risk of cirrhotic decompensation and mortality in patients with cirrhosis, while in the non-cirrhotic patients, statins were not significantly associated with the risk of developing cirrhosis or fibrosis progression (104). However, Athyros et al. suggested a different view. They suggested that statin therapy might play a role in reducing steatosis and improving steatohepatitis and fibrosis (105). A large population-based nested case-control study backed up their point. Statin could reduce NAFLD risk and liver fibrosis risk in NAFLD patients (106). Another four-year study reported that the administration of atorvastatin at a dose of 20 mg in combination with vitamins could effectively reduce the odds of developing hepatic steatosis by 71% in NAFLD patients (107). Similarly, the oral administration of rosuvastatin at a dose of 5 mg/d for 24 weeks could also reduce LFC in patients (108).

However, it has long been generally accepted that statin is potentially hepatotoxic; therefore, its use for the treatment of NAFLD patients has been low in the past. A recent analysis of trends in statin use among adults with NAFLD in the United States suggested that the onset of NAFLD was associated with inadequate early statin use in the disease (109). With the in-depth study of statin, this trend is gradually changing. Studies also showed that statin was associated with a significant reduction in cancer-related mortality in NAFLD patients (110). NAFLD/NASH patients often have a high CVD risk. Studies showed that the application of statins could significantly reduce the CVD risk of NAFLD/NASH patients (111). In a randomized controlled trial, evaluating the effects of pioglitazone in the treatment of NASH, a *post hoc* analysis of the statin safety showed that statin could reduce plasma total cholesterol levels and LDL-C levels. The three-year follow-up reported no adverse effect of statin on liver enzyme levels; therefore, they suggested that it could be used in T2DM patients with NAFLD (112). On the other hand, the analysis of statin use and risk of new-onset diabetes indicated that statins were associated with a risk of hyperglycemia and their use should be limited in patients with prediabetes or at risk for diabetes (113).

Ezetimibe, a cholesterol absorption inhibitor, could significantly improve fatty liver in rats by increasing cholesterol efflux transporter (114) and could also prevent and improve liver steatosis in the HFD mice (115). Ezetimibe at a dose of 10 mg/d for 6 months could significantly reduce serum total cholesterol levels and improve liver fibrosis scores in NAFLD patients (116). In a study by Park et al., a similar treatment regimen for 24 months could improve metabolism and liver histology in NAFLD patients, showing promising results (117). In another study, a treatment regimen, containing 10 mg/d of ezetimibe and 5 mg/d of rosuvastatin for 24 weeks, could significantly reduce LFC in patients (108). However, a meta-analysis by Lee et al. showed that ezetimibe could not improve liver steatosis (118). Another study also claimed that in combination with lifestyle intervention, ezetimibe could not improve liver histology in NASH patients (119). Therefore, further studies are needed to demonstrate its efficacy.

#### 2.2.3 Antihypertensive drugs

An increase in intrahepatic vascular resistance has been observed in both the early-stage NAFLD patients and animal models of NAFLD (120, 121), leading to tissue hypoxia and triggering disease progression. The liver vessels are highly responsive to vasoconstrictors. Clinically, NAFLD is associated with tew-onset hypertension in patients, and elevated blood pressure is associated with the progression of fatty liver disease and fibrosis in patients. Therefore, vasoconstrictor antagonists might be used for the treatment of NAFLD (122, 123).

In the Wistar rat model of diet-induced steatosis, endothelin-1 (ET-1), angiotensin II (AT-II), thromboxane A2 (TxA2) could increase the transhepatic pressure gradients (THPG), which could be reduced by bosentan (ET-1 antagonist), valsartan (AT-II-receptor blocker) and celecoxib (COX-2 inhibitor). In the Zucker rat model of NASH, comparing the therapeutic effects of bosentan and placebo showed that bosentan could reduce steatosis and structural liver damage; therefore, the vasoconstrictor antagonists might improve intrahepatic vascular function, demonstrating their potential therapeutic effects in early NAFLD (122). In the diet-induced rat model of NASH, the combination of atorvastatin and ambrisentan could significantly improve liver histology, thereby suggesting the potential application of combination therapy in NASH patients (124). In the NAFLD mouse models, Losartan could prevent hepatic steatosis and macrophage polarization in the ob/ob mice by inhibiting the hypoxia-inducible factor-1α (HIF-1α). The Losartan-treated mice showed a significant increase in the levels of TG and free fatty acid (FFA), thereby preventing NAFLD (125). Telmisartan, also a potential therapeutic drug for NAFLD, could inhibit liver fibrosis by blocking the AT-II receptors (126). Studies also showed that it was a partial agonist of PPAR-γ, thereby improving IR, lipid metabolism and disease condition of liver steatosis and fibrosis and reducing the expression of pro-inflammatory cytokines as well as the levels of fatty acids and TGs (127). In general, based on animal experiments, antihypertensive drugs like sartans have shown efficacy in the treatment of NAFLD; however, their similar effects in NAFLD patients require clinical verification.

#### 2.2.4 Hepatoprotective drugs

UDCA is an endogenous synthetic bile acid in the human body and has antioxidant and anti-inflammatory effects, preventing mitochondrial dysfunction in the progression of obesity-related diseases. In some early clinical trials, UDCA did not show much efficacy in the NAFLD/NASH patients (128–130). However, its liver protective effects had been demonstrated; therefore, both animal and clinical studies on UDCA did not stop. In ob/ob mice, UDCA could regulate liver energy homeostasis and macrophage polarization in the white adipose tissues (131). A randomized controlled trial by Ratziu et al. suggested that the high-dose (28-35 mg/kg/d) UDCA treatment for 12 months could reduce hepatic aminotransferase levels in NASH patients and improve glycemic control and IR. In addition, UDCA has a good safety profile with no deterioration of liver function, and the main adverse reactions are abdominal discomfort and diarrhea (132). A study by Nadinskaia et al. supplemented UDCA at 15 mg/kg/d based on changes in diet and exercise. In the first three months, NAFLD patients showed normalization of the liver enzyme levels and improvement in lipid profile and fatty liver degeneration, and the six-month treatment was beneficial to reduce CVD risk (133).

Farnesol X receptors (FXR), a member of the nuclear receptor superfamily, regulate bile acid synthesis, glycolipid metabolism, liver inflammation, and fibrosis processes. The FXR agonists enhance insulin sensitivity, inhibit bile acid synthesis, and promote mitochondrial fatty acid oxidation. Obeticholic acid (OCA) is a representative FXR drug (134). In a phase II trial by Sunder Mudaliar et al., a continuous treatment with 25 or 50 mg/d OCA for 6 weeks could improve IR and reduce the levels of liver inflammatory and fibrosis markers in the T2DM patients with NAFLD and was well-tolerated (135). In a double-blind placebo-controlled trial by Neuschwander-Tetri et al., the 25 mg/d OCA treatment for 72 weeks could improve the histological features of NASH; however, the long-term OCA administration resulted in pruritus in about 23% of patients (136). Based on previous studies, Younossi et al. designed a phase III clinical trial to evaluate the effects of OCA on fibrosis in NASH patients (137). They conducted an interim analysis after 18 months of treatment and showed that 10 or 25 mg/d OCA treatment could significantly improve fibrosis in NASH patients; however, long-term OCA treatment could cause skin and subcutaneous tissue diseases, gastrointestinal disorders, and cholesterol (138). These adverse reactions might limit the use of OCA in NAFLD/NASH patients.

#### 2.2.5 Probiotics/prebiotics/synbiotics

Targeting intestinal microbes has been a hot topic in NAFLD therapy in recent years. Gut microbiota might play a role in the pathogenesis of NASH by releasing lipopolysaccharide (LPS), increasing ethanol production, and activating inflammatory cytokines in the luminal epithelial cells and hepatic macrophages (139). In addition, the gut microbiota can also influence the development of NAFLD by increasing the metabolisms of choline and bile acids and the production of SCFAs (acetate, propionate, and butyrate) during bacterial fermentation (140). Moreover, gut dysbiosis, such as the abnormal release of LPS and SCFAs and ceramides fibrosis, induces liver inflammation and hepatic inflammation by increasing the release of damage-associated molecular patterns (DAMPs) and pathogen-associated molecular patterns (PAMPs) (141, 142). In the mice’s gut, the reduced butyrate levels and increased bacterial LPS translocation could contribute to NAFLD and IR. However, its effectiveness in clinical applications has not been explored yet. Generally, intestinal microecological disorders are prevalent in chronic liver diseases and play an important role in the occurrence and development of liver cirrhosis caused by various etiologies (143). Probiotics, prebiotics, synbiotics, fecal microbiota transplantation (FMT), etc. are increasingly appearing in clinical trials as common intestinal microecological regulatory methods (144).

In a placebo-controlled trial by Aller et al., the oral combination of *Lactobacillus bulgaricus* and *Streptococcus thermophilus* at 500 million CFU/d for 3 months could improve liver aminotransferase levels in patients with NAFLD (145). A randomized controlled trial showed that as compared to milk, the probiotic-fortified yogurt was more beneficial in improving IR and LFC in obese women with NAFLD and MS. A daily intake of 220 g of yogurt for 24 weeks could reduce inflammation and oxidative stress, modulate lipid metabolism, and alter the composition of gut microbiota (146). Based on diet and exercise intervention, the supplementation of *Saccharomyces boulardii* powder (Beda Pharma, France) for 12 weeks could significantly decrease the levels of total bilirubin (TBil), AST, ALT, GGT, serum total cholesterol, LDL-C, and fasting insulin in NAFLD patients (147). Another placebo-controlled trial suggested that the oral administration of probiotic capsules Lactocare (Zist-takhmir, Tehran, Iran, containing 7 kinds of probiotics) at a dose of 2 capsules/d for 8 consecutive weeks could improve IR in NAFLD patients and decrease the levels of inflammatory factors, including tumor necrosis factor α (TNF-α) and interleukin- 6 (IL-6) (148). Similarly, the probiotic Symbiter (Prolisok, Ukrainian, a mixture of 14 probiotics) could reduce the levels of LFC, TNF-α, and IL-6 and transaminase activity in patients with NAFLD (149). In another study, the oral supplementation of Symbiter once per day with omega-3 fatty acids (250 mg each of edible flax and wheat germ oil, the omega-3 fatty acid content of 3%-5%) for 8 weeks could reduce LFC in NAFLD patients, improve blood lipid content, metabolic level, and reduce systemic chronic inflammation (150). However, some studies have reported different results. Recently, in a randomized, double-blind, placebo-controlled trial, the oral administration of a multi-strain probiotic, containing six different *Lactobacillus* and *Bifidobacterium* species (MCP BCMC strains), at 30 billion CFU/d for 6 months showed no significant effects on hepatic steatosis and fibrosis. However, this study indicated that probiotics could stabilize the immune function of the intestinal mucosa and improve mucosal permeability (151).

A placebo-controlled trial of 14 NASH patients reported that the oral administration of fructo-oligosaccharides (Fos) (8 g/d for 12 weeks, then 16 g/d for 24 weeks) could reduce histological steatosis in NASH patients (152). Besides, based on the changes in diet and exercise, the oral administration of *Bifidobacterium longum* and Fos for 24 weeks could significantly reduce serum TNF-α, CRP, and AST levels in NASH patients, and improve liver steatosis and fibrosis score (153). Similarly, another placebo-controlled trial also suggested that synbiotics could improve TNF-α and CRP levels in NAFLD patients (154). A double-blind placebo-controlled phase II trial, including 104 NAFLD patients, showed that the oral administration of synbiotics (*Bifidobacterium animalis* and Fos) for one year could alter the composition of gut microbiota, containing high proportions of *Bifidobacterium* and low proportion of *Oscillibacter* and *Alistipe* species, but did not alter LFC or fibrosis. In another placebo-controlled study, the supplementation of probiotic formulations Familact (Zisttakhmir, containing seven probiotics) and Fos for 8 weeks could improve liver steatosis in NAFLD patients; however, it had no significant effects on liver enzyme levels (155).

An FMT trial by Craven et al. enrolled 21 patients with a 6-month follow-up. The results suggested that allogeneic or autologous FMT could not significantly improve IR in NAFLD patients, but could potentially improve small intestinal permeability (156). Recently, Chinese researchers conducted an FMT and oral probiotics-based randomized controlled clinical trial. The FMT group patients were orally administered with 200 mL of fresh bacterial solution for three consecutive days, and the probiotic group patients were orally administered with *Bifidobacterium* preparations and *Lactobacillus acidophilus* capsules. Both the groups had a daily healthy diet and performed over 40 minutes of exercise. The results of a one-month follow-up showed that FMT could reduce fat accumulation in the liver by improving the dysbiosis of gut microbiota, thereby reducing fatty liver disease and showing more effectiveness in non-obese NAFLD patients (157).

In general, most of these trials suggested that targeting the gut microbiota might have a certain therapeutic effect on NAFLD. However, related studies have come to different conclusions. There is no uniform standard for the time of intervention, a method used, number of samples, indicators of the study, and endpoint of the study. Therefore, further optimization of the study is needed to evaluate the real benefits of probiotics in the treatment of NAFLD.

#### 2.2.6 Peroxisome proliferation-activated receptor agonist

PPAR agonists can help in improving blood sugar and lipid levels (fat and cholesterol). In addition to the T2DM drug pioglitazone mentioned above, numerous PPAR agonists are currently being developed. Saroglitazar, a dual PPRA-α/γ agonist, can regulate glucose and lipid metabolism and is currently approved in India as an NAFLD therapeutic drug (158). A study by Indian scholars showed that Saroglitazar could significantly improve hepatic steatosis in high-fat diet-induced NASH male Wistar rats (159). A randomized, double-blind, controlled phase II clinical trial conducted by Gawrieh et al. found that the 4-mg/d dose of Saroglitazar in NAFLD/NASH patients for 16 weeks could improve dyslipidemia and significantly reduce fat contents and triglyceride levels in the liver while improving IR (160). A randomized, double-blind, placebo, controlled phase IIb clinical trial in NASH patients revealed that the 1200-mg/d dose of Lanifibranor, a pan-PPAR agonist, for 24 weeks could significantly reduce the patient’s liver fibrosis score (SAF-A score), decrease liver lipid content, and improve liver enzyme levels, and Francque’s team will conduct a phase III trial of the drug for further validation (161). A clinical study by Gastaldelli’s and colleagues also showed that the PPAR-γ agonist pioglitazone could induce changes in visceral fat and adiponectin levels, thereby alleviating steatohepatitis in NASH patients (162). Ratziu et al. showed that the 120-mg/d dose of Elafibranor, a PPAR-α/δ agonist, for one year significantly improved the fat content in the liver as well as the liver enzyme levels without worsening fibrosis (163). A clinical trial conducted by Japanese scholars found that although Pemafibrate, a novel selective PPAR-α modulator, could not significantly reduce fat contents, it significantly reduced liver stiffness and was recommended to treat NASH in combination with other drugs (164). In conclusion, the overall safety and tolerability of these drugs are good; however, the minor adverse effects, which are currently faced by patients, also require further investigations (**Table 3**).

**Table 3:** Effect of PPRA agonist in patients of NAFLD/NASH: Data from clinical studies.

	Drugs	Types	Treatment duration	Population	Effects	Adverse events	References
PPRA-γ agonist	Pioglitazone (45mg/day) and additional dietary and exercise interventions	randomized, double-blind, placebo-controlled trial	18 mouths	T2DM with NASH	improvement in liver histology, not worsened fibrosis	weight gain (in women)	Cusi et al. (71)
	Pioglitazone(30mg/day)	randomized, controlled clinical trial	96 weeks	NASH	improvement in liver histology, liver enzyme level and IR	weight gain	Sanyal et al. (98)
	Pioglitazone (30mg/day)	randomized placebo-controlled trial	12 mouths	NASH	improvement in Liver metabolic and histological parameters	rash, fluid retention	Aithal et al. (99)
	Pioglitazone (45mg/day) and additional dietary and exercise interventions)	placebo-controlled trial	6 mouths	impaired glucose tolerance or T2DM and NASH	improvement in metabolism and liver histology	Fatigue and mild lower-extremity edema	Belfort et al. (100)
PPRA-α/γ agonist	Saroglitazar(1/2/4 mg/day)	randomized, double-blind, controlled phase 2 clinical trial	24 weeks	NASH	liver histology improvement	NA	Siddiqui et al. (158)
	Saroglitazar (1/2/4 mg/day)	randomized, double-blind, controlled phase 2 clinical trial	16 weeks	NAFLD/NASH	liver fat content and triglyceride levels improvement	diarrhea, cough, abdominal pain, bronchitis and rash	Gawrieh et al. (160)
Pan-PPRA agonist	Lanifibranor (800/1200 mg/day)	double-blind, randomized, placebo-controlled phase 2b trial	24 weeks	NASH	fibrosis score (SAF-A) improvement	diarrhea, nausea, peripheral edema, anemia, and weight gain	Francque et al. (161)
PPRA-α/δ agonist	Elafibranor (80/120 mg/day)	international, randomized, double-blind placebo-controlled trial	52 weeks	NASH	LFC and liver enzyme improvement, not worsened fibrosis	reversible increase in creatinine levels	Ratziu et al. (163)
PPRA-α agonist	Pemafibrate (0.2mg twice daily)	double-blind, placebo-controlled, randomised multicentre, phase 2 trial	72 weeks	NASH	liver hardness decrease	gastrointestinal disorders, cough, rash	Nakajima et al. (164)

#### 2.2.7 Thyroid hormone receptor β (THR-β) agonist

THR-β is involved in various metabolic pathways, such as glycolipid and cholesterol metabolisms in the liver, and has also been used as a therapeutic target for the research and development of novel therapeutic drugs for NAFLD/NASH. Resmetirom (MGL-3196), a novel THR-β agonist, targets the liver. The treatment of high-fat and high-sugar diet-induced obese NASH mice with MGL-3196 treatment at a 3-mg/kg/day dose could significantly reduce liver weight, hepatic steatosis, plasma ALT activity, liver and plasma cholesterol, and blood glucose (165). A 36-week randomized, double-blind, placebo-controlled, multicenter phase II clinical trial conducted by Harrison’s team showed significant reductions in the liver fat contents in NASH patients after 12 and 36 weeks of oral treatment with 80 mg/day of MGL-3196 (166). In an expansion study of this phase II study, the oral treatment with 80 or 100 mg/d of MGL-3196 was well-tolerated and safe for patients, and the drug is currently in phase III clinical study (167). TG68 is another novel THR-β agonist used to treat NAFLD. In animal studies, TG68 has shown significant effects on the liver fat contents and hepatic steatosis in high-fat diet-induced NASH C57BL/6 mice, and its efficacy is comparable to that of MGL-3196 (168); however, further clinical studies are needed to verify these results.

#### 2.2.8 Fibroblast growth factor analogs

FGF19 and FGF21 are novel endocrine messengers, which regulate multiple aspects of energy homeostasis, such as lipid and carbohydrate metabolisms. Their analogs were initially developed to improve hyperglycemia in T2DM patients; however, their robust, consistent, and durable effects on lipid metabolism in human trials gradually transformed their clinical emphasis toward their use for NASH treatments. Therefore, they are now considered credible and promising novel NAFLD/NASH therapeutic drugs (169). In a randomized, double-blind, placebo-controlled Phase II clinical study, the treatment of NASH patients with 3 or 6 mg/day of Aldafermin (NGM282), an engineered analog of the gut hormone FGF19, reduced liver fat content with a good safety profile (170). A multicenter, randomized, double-blind, placebo-controlled study conducted by Harrison’s team revealed that the treatment of NASH patients with 1 or 3 mg/d of NGM282 improved the histological features and fibrosis score of NASH over 12 weeks while also improving the levels of liver enzymes, such as AST and ALT (171). Subsequently, they conducted a 24-week phase II clinical study, which also showed that the NGM282 treatment reduced liver fat and tended to improve fibrosis (172). Recently, their team conducted a randomized, double-blind, placebo-controlled, phase IIb clinical study, in which, they set up different doses of the drug groups, including 0.3 mg/d and 1 mg/d. The results showed that the treatment was generally well-tolerated; however, there was no significant difference between the different doses in improving fibrosis (173). Studies also showed that NGM282 could reduce liver fat, liver damage, and inflammation in NASH patients, but elevated cholesterol levels. In a multicenter, open-label phase II clinical study, in combination with statins, NGM282 was more effective in controlling cholesterol levels (174). Overall, NGM282 is well-tolerated and safe, and the common adverse reactions include nausea, vomiting, and mild or moderate diarrhea.

FGF21, a member of the FGF19 signaling subfamily, is considered a potential target for the treatment of NAFLD. Targeting the FGF21/FGFR/β-Klotho signaling pathway might block or reverse hepatic fat infiltration, inflammation, and fibrosis (175). Pegbelfermin (BMS-986036) is a PEGylated FGF21analogue. The phase II clinical studies in obese and T2DM patients suggested that BMS-986036 could improve metabolic parameters and fibrotic markers (176). A multicenter, randomized, double-blind, placebo-controlled phase IIa study showed that the subcutaneous injection of NASH patients with 10 mg/day or 20 mg/week of BMS-986036 for 16 weeks reduced the liver fat scores and was well-tolerated by the patients (177). There are currently fewer clinical studies on these drugs, and more clinical trials are needed to confirm their long-term benefits on the outcomes, such as liver histology, development of cirrhosis, or survival (178).

#### 2.2.9 Others

TVB2640 is a novel lipase synthesis inhibitor, which can reduce liver fat synthesis in men (179). A phase IIa clinical trial suggested that the treatment of patients with TVB2640 at the doses of 25/50 mg/day for 12 weeks significantly reduced liver fat contents and improved the biomarkers of liver biochemistry, inflammation, and fibrosis in a dose-dependent manner (180). Namodenoson, an A3 adenosine receptor (A3AR) agonist, also showed promising results in a recent phase II clinical trial by improving the liver function/pathology in NASH (181). Currently, other novel drugs are being investigated in clinical studies, such as HM15211, a novel long-acting GLP-1/GIP/Glucagon triple agonist; AZD2693, a Patatin-like phospholipase domain containing 3 (PNPLA3) antisense oligonucleotide drug; and BMS-986263, a lipid nanoparticle drug containing siRNA that inhibits heat shock protein 47 (HSP47). Some new drugs for NAFLD/NASH patients, which are in clinical trials, are summarized in **Table 4**.

**Table 4:** Partial drug clinical trials in progress or in preparation (data from ClinicalTrials.gov).

	Drugs	Population	Clinical trial registration number	Phase of clinicial trail	Status
GLP-1RAs	Semaglutide	NASH	NCT04822181	3	Recruiting
		T2DM with NAFLD	NCT05067621	3	Not yet recruiting
	Semaglutide and NNC0194-0499(a new medicine which works in the liver) /NNC0174-0833 (a novel weight-loss medicine)	NASH	NCT05016882	3	Recruiting
	Tirzepatide (LY3298176)	NASH	NCT04166773	2	Recruiting
PPRA--α/γ agonist	Lanifibranor	T2DM with NASH	NCT03459079	2	Recruiting
		NASH	NCT04849728	3	Recruiting
THR-β agonist	MGL-3196	NAFLD	NCT04951219	3	Recruiting
		NASH	NCT03900429	3	Recruiting
		NASH	NCT02912260	2	Unknown
Fatty acid synthase inhibitors	TVB-2640	NASH	NCT04906421	2	Active, not recruiting
A3AR	Namodenoson	NASH	NCT04697810	2b	Recruiting
GLP-1/GIP/Glucagon triple agonist	HM15211	NASH	NCT04697810	2	Recruiting
Oligonucleotide drugs targeting Patatin-like PNPLA3 gene	AZD2693	NASH	NCT04166773	2	Recruiting
lipid nanoparticle drug containing siRNA that inhibits HSP47	BMS-986263	NASH	NCT04267393	2	Recruiting
novel insulin sensitizer target mitochondrial pyruvate carriers	MSDC-0602K	T2DM with NAFLD/NASH	NCT03970031	3	Recruiting
β-Klotho/FGFR1c receptor agonist	MK-3655 (NGM313)	NASH	NCT04583423	2b	Recruiting

## 3 Summary and outlook

NAFLD, a multi-system metabolic disease, is usually accompanied by T2DM and MS, which jointly promote disease progression. The incidence of NAFLD is increasing annually, and it has a worldwide prevalence, while its pathogenesis has not been fully understood yet. Therefore, NAFLD has become a new challenge and a major public health concern in the field of liver diseases and metabolisms worldwide. Lifestyle intervention is an important basic treatment. Regulating glucolipid metabolism has still been focused on in the NAFLD treatment. The studies on targeting gut microbiota for the treatment of NAFLD are also deepening. Moreover, various novel metabolic drugs represented by PPAR agonists, FXR agonists, and THR-β agonists are also being developed and are in the clinical trial stages. However, developing new drugs for NAFLD has still a long way to go. The results of clinical trials of most therapeutic drugs are not ideal, and the liver histological endpoint has not been reached yet, which might be due to the complex heterogeneity of NAFLD, limitations of therapeutic targets, drug safety, and other factors. An in-depth understanding of the pathogenesis of NAFLD might help in. finding drugs, which can not only improve metabolic disorders but also reduce liver inflammation and fibrosis in the near future. Of course, considering the medical, economic, and social burden caused by the global epidemic of NAFLD, the global vulnerable population, medical personnel, and medical institutions should improve their awareness of this disease in order to make early interventions and achieve better prevention and control effects.

## Author contributions

LR and JZ jointly conceived and drafted the review. WR and XQ suggested some important changes to the content of the review. YC and HC refined the language, and JG directed and proofread the review throughout. All authors contributed to the article and approved the submitted version.
